# Protein modeling and clinical description of a novel in‐frame *GLB1* deletion causing GM1 gangliosidosis type II

**DOI:** 10.1002/mgg3.454

**Published:** 2018-09-05

**Authors:** John E. Richter, Michael T. Zimmermann, Patrick R. Blackburn, Ahmed N. Mohammad, Eric W. Klee, Laura M. Pollard, Colleen F. Macmurdo, Paldeep S. Atwal, Thomas R. Caulfield

**Affiliations:** ^1^ Department of Clinical Genomics Mayo Clinic Jacksonville Florida; ^2^ Center for Individualized Medicine Mayo Clinic Jacksonville Florida; ^3^ Division of Biomedical Statistics and Informatics Department of Health Sciences Research Mayo Clinic Rochester Minnesota; ^4^ Center for Individualized Medicine Mayo Clinic Rochester Minnesota; ^5^ Department of Laboratory Medicine and Pathology Mayo Clinic Rochester Minnesota; ^6^ Biochemical Genetics Laboratory Greenwood Genetic Center Greenwood South Carolina; ^7^ Division of Medical Genetics Baylor Scott & White Health Temple Texas; ^8^ Department of Neuroscience Mayo Clinic Jacksonville Florida

**Keywords:** Beta‐galactosidase‐1 (GLB1), GM1 gangliosidosis type II, molecular modeling

## Abstract

**Background:**

Beta‐galactosidase‐1 **(** GLB1) is a lysosomal hydrolase that is responsible for breaking down specific glycoconjugates, particularly GM1 (monosialotetrahexosylganglioside). Pathogenic variants in *GLB1* cause two different lysosomal storage disorders: GM1 gangliosidosis and mucopolysaccharidosis type IVB. In GM1 gangliosidosis, decreased β‐galactosidase‐1 enzymatic activity leads to the accumulation of GM1 gangliosides, predominantly within the CNS. We present a 22‐month‐old proband with GM1 gangliosidosis type II (late‐infantile form) in whom a novel homozygous in‐frame deletion (c.1468_1470delAAC, p.Asn490del) in *GLB1* was detected.

**Methods:**

We used an experimental protein structure of β‐galactosidase‐1 to generate a model of the p.Asn490del mutant and performed molecular dynamic simulations to determine whether this mutation leads to altered ligand positioning compared to the wild‐type protein. In addition, residual mutant enzyme activity in patient leukocytes was evaluated using a fluorometric assay.

**Results:**

Molecular dynamics simulations showed the deletion to alter the catalytic site leading to misalignment of the catalytic residues and loss of collective motion within the model. We predict this misalignment will lead to impaired catalysis of β‐galactosidase‐1 substrates. Enzyme assays confirmed diminished GLB1 enzymatic activity (~3% of normal activity) in the proband.

**Conclusions:**

We have described a novel, pathogenic in‐frame deletion of *GLB1* in a patient with GM1 gangliosidosis type II.

## 1 INTRODUCTION

*GLB1* (MIM: 611458) is located on chromosome 3 and gives rise to two alternatively spliced mRNA transcripts that encode beta‐galactosidase‐1 (GLB1) and the elastin‐binding protein (EBP) (Privitera, Prody, Callahan, & Hinek, 2008). β‐galactosidase‐1 is a lysosomal hydrolase responsible for cleaving terminal beta‐galactose residues from ganglioside substrates and other glycoconjugates (Yoshida et al., 2004). Pathogenic alterations in *GLB1* lead to a reduction in affinity for at least one substrate, resulting in either the toxic accumulation of GM1 ganglioside in the nervous system leading to progressive neurological deterioration (GM1 gangliosidosis) or defective storage of galactosyl oligosaccharides and keratin sulfate causing mucopolysaccharidosis type IVB, Morquio type (MIM: 253010) (Regier & Tifft, 2005). In GM1 gangliosidosis, defective β‐galactosidase‐1 causes cell lysosomes to accumulate GM1 ganglioside and other glycoconjugates over time—a process that has the most pronounced effects on the central nervous system (CNS) since gangliosides are particularly abundant in the neuronal plasma membrane (Posse de Chaves & Sipione, 2006). This accumulation leads to neuronal cell death and progressive CNS dysfunction with variable severity and age at onset (Brunetti‐Pierri & Scaglia, 2014).

GM1 gangliosidosis is divided into three types that generally correlate with residual enzyme activity, of which type I (MIM: 230500) is the most severe and has the earliest onset (Regier and Tifft, 2005). Type I symptoms appear before 1 year of age and present with CNS dysfunction, psychomotor regression, “coarse” facial features, cardiac involvement, skeletal abnormality, macular cherry‐red spots, and hepatosplenomegaly (Caciotti et al., 2017). Type II (MIM: 230600) symptoms can appear between 7 months and 3 years of age (late infantile) or 3–10 years of age (juvenile), and include progressive motor abnormalities, muscle weakness, seizures, strabismus, corneal clouding, and diffuse brain atrophy (Caciotti et al., 2017; Regier and Tifft, 2005). Cardiac involvement, skeletal abnormalities, and hepatosplenomegaly can also be seen, though typically not to the extent seen in type I (Caciotti et al., 2017). Type III (MIM: 230650) usually presents between 3 to 30 years of age with slurred speech, dystonia, parkinsonism, cerebellar dysfunction, and mild vertebral deformity and is the mildest form (Caciotti et al., 2017). In practice, distinguishing between type I and II GM1 gangliosidosis can be challenging given their overlapping clinical presentation and age of onset, a difficulty not encountered with type III cases (Regier & Tifft, 2005). Features that can be used to distinguish between type I and II GM1 gangliosidosis include the presence of cherry‐red macula and placental storage of GM1 ganglioside in type I cases (Roberts, Ampola, & Lage, 2015).

In this report, we describe a patient presenting with phenotypes most consistent with a diagnosis of GM1 gangliosidosis type II. Sequencing revealed the presence of a novel homozygous in‐frame deletion in *GLB1*. We performed detailed structural modeling and molecular dynamic simulations to determine the impact of this variant on GLB1 protein function. In addition, an enzyme assay showed reduced β‐galactosidase‐1 activity, thus supporting our structural analyses.

## 2 CLINICAL DESCRIPTION

The proband initially presented at 22 months old with the tentative diagnosis of Hurler syndrome (mucopolysaccharidosis Ih, MIM: 607014). Her constellation of symptoms first began to appear upon her premature delivery at 26 weeks of gestation. At birth, the proband had retinopathy of prematurity leading to blindness, apnea, congenital gastroschisis, gastroesophageal reflux disease, oral aversion, a left ovarian cyst, a ventricular septal defect (VSD), patent ductus arteriosus (PDA), and dysphagia. Subsequently, she developed bronchopulmonary dysplasia due to respirator use and later on experienced an intraventricular hemorrhage with sequela of tonic–clonic seizures. Several corrective surgeries were performed including a gastroschisis closure, Nissen fundoplication, gastrostomy for oral aversion and dysphagia, left laparoscopic oophorectomy, PDA ligation, and VSD repair.

Before her arrival at our clinic, brain MRI and spinal X‐ray had been performed. The brain MRI revealed encephalomalacia of the inferior cerebellar hemispheres consistent with the proband's intraventricular hemorrhage, borderline inadequate myelination patterns, and bilateral mastoid effusions. The spinal X‐rays showed gibbus deformity with thoracolumbar kyphosis and scoliosis and AP foreshortening with inferior anterior beaking at L2‐L5. When the proband was first seen by genetics, she displayed failure to thrive, motor regression, and stiffening of joints in addition to the symptoms noted above. Her physical examination was notable for abnormally flat facial profile, small mouth and prominent tongue, gingival hyperplasia, hepatomegaly, generalized hypotonia, shortened limbs, and nonambulatory gait requiring bilateral ankle‐foot orthotic braces. A lysosomal enzyme panel was performed for clinical suspicion of Hurler syndrome.

While alpha‐L‐iduronidase activity was normal, the proband was found to have 3% of normal beta‐galactosidase‐1 activity. Subsequent *GLB1* sequencing revealed that the proband had a homozygous deletion in *GLB1*, denoted c.1468_1470delAAC [Chr3(GRCh37): g.33058210_33058212del, NM_000404.3: c.1468_1470del, NP_000395.2: p.Asn490del]. This alteration has been observed rarely in gnomAD (rs780232995, allele count: 2/277218, MAF: 0.000007215%; Latino population allele count: 2/34420, MAF: 0.00005811%) (Lek et al., 2004). A clinical microarray (Affymetrix CytoScan HD) confirmed that there were no other deletions, duplications, or copy number variations of clinical significance. Additionally, no long contiguous stretches of homozygosity were reported by microarray. The mechanism whereby p.Asn490del impacts GLB1 enzymatic function is unknown. Other similar in‐frame deletions in *GLB1* have been reported; however, they fall in other regions of the protein (Caciotti et al., 2017; Santamaria, Blanco, Chabás, Grinberg, & Vilageliu, 2016; Yang, Wu, & Tsai, 1996). In order to determine the effects of this mutation, we performed molecular dynamics simulations comparing the mutant and wild‐type (WT) protein.

**Figure 1:**
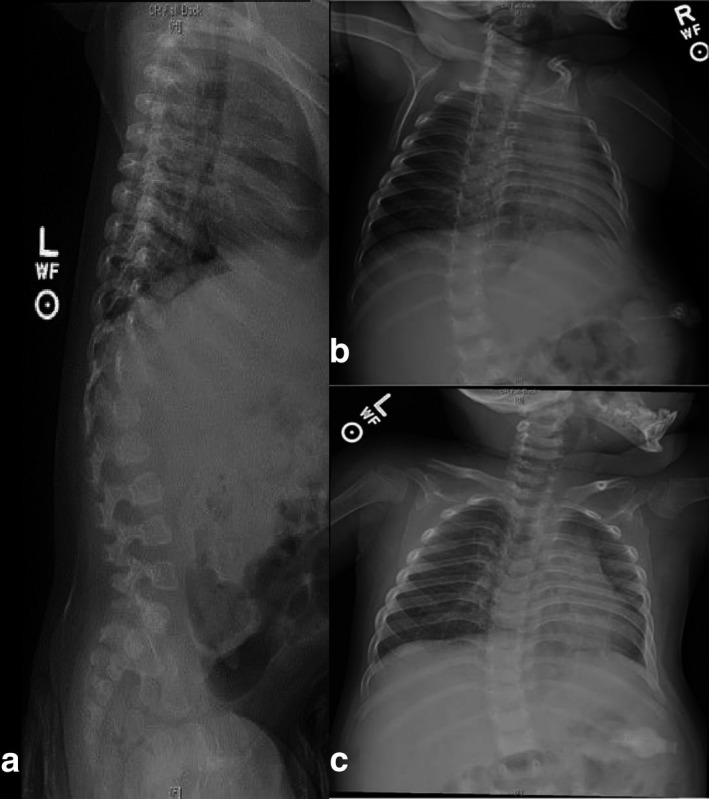
Osseous survey of the proband taken at 22 months of age showing dysostosis multiplex. (a) Thoracolumbar gibbus deformity, mild AP foreshortening with inferior anterior beaking at L2‐L5 (L3 is the least affected). (a–c) Abnormal hypoplastic vertebral bodies with foreshortening and rounded appearance of the endplates at T11, T12, and L1, and to a lesser extent at T10. (b‐c) The medullary cavity of the proximal humeral diaphysis bilaterally appears expanded with relatively thin cortices

Following genetic testing, an osseous survey detected multiple abnormalities in the proband consistent with dysostosis multiplex (Figure 1). The proband was ultimately diagnosed with GM1 gangliosidosis type II, though her presenting hepatomegaly, coarse facial features, and gingival hyperplasia are more typical of type I. It should be noted that the types of GM1 gangliosidosis overlap in their phenotypic spectrum. Her enzyme assay detected ~3% (0.49 nmol/hr/mg, normal range 13.5‐176) β‐galactosidase activity which falls in the range expected for late‐infantile type II patients at 1%–5% of normal activity. Type I GM1 gangliosidosis typically exhibit negligible levels of β‐galactosidase activity (Suzuki et al., 2017). Continued multispecialty follow‐ups were recommended in order to monitor the disease's progression and treat the proband's symptoms.

## 3 METHODS

We used the experimental structure of GLB1 (PDB ID 3thc) (Ohto et al., 2016) in molecular modeling. Loops that were unresolved in the experimental structure were added (Eswar et al., 2014) and a model of p.Asn490del was generated. We used implicit solvent molecular dynamics (Generalized Bourne with Simple Switching (Chocholousova & Feig, 2006; Feig et al., 2012) as implemented in (Discovery Studio Modeling Environment, Release 2017, San Diego: Dassault Systèmes, 2011), the CHARMM36 force field, an interaction cutoff of 12Å, with strength tapering beginning at 10Å, a simulation time step of 1 fs, and conformations recorded every 2 ps. To focus our simulations on the ligand‐binding site and catalytic residues, large interdomain motions were restrained using harmonic constraints. GLB1 was simulated with bound beta‐D‐galactose as described in the ZINC database (Irwin & Shoichet, 2008) (ID 2597049). Each initial conformation was used to generate 5 replicates. Each replicate was independently energy minimized for 2,000 steps by steepest descent, minimized for 2,000 steps by conjugate gradient, heated to 300K over 300 ps via a Langevin thermostat, and simulated for 12 ns. One replicate of each sequence was extended for an additional 10 ns and without constraints. We used VMD for molecular visualization (Humphrey, Dalke, & Schulten, 2016).

## 4 RESULTS

**Figure 2:**
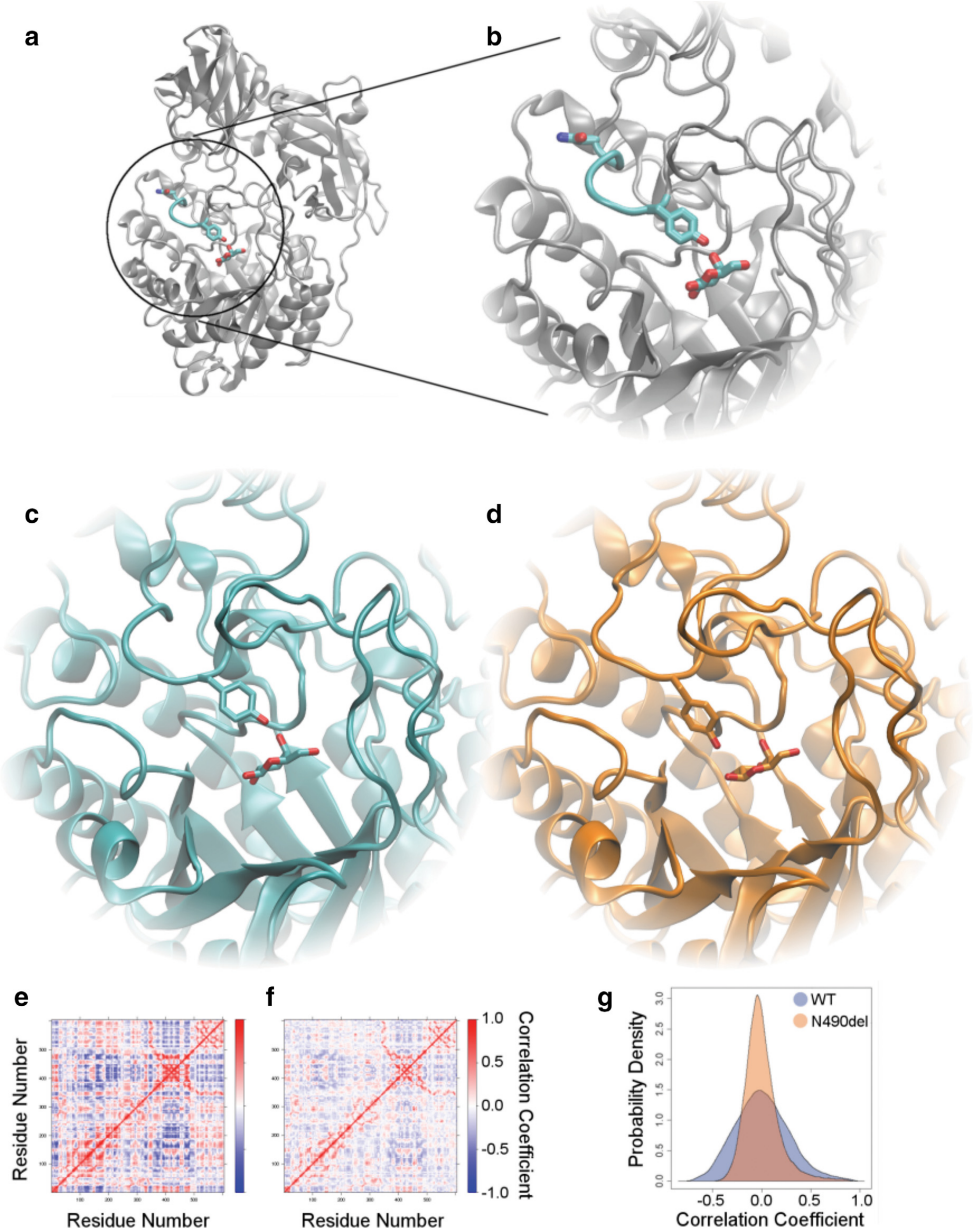
Novel in‐frame deletion affects ligand binding to GLB1. a) The structure of GLB1 is shown with Asn490, Tyr485, and ligand shown in detail. The residues connecting Tyr485 to Asn490 are highlighted and b) shown zoomed in. c) After simulation, WT maintains the same interaction between ligand and Tyr485, while d) Asn490del showed altered interaction. e) In unconstrained simulations, we monitored the correlation between each residue's motions and show these pairwise correlations as a matrix. Certain regions of the structure tend to move in a coordinated way, corresponding to interdomain motions. f) Asn490del shows the same type of motion, but with less coordination. g) The same correlation data are shown as a density plot to emphasize the loss of strong correlations

Asn490 is located within a ligand‐binding loop and the nearby Tyr485 makes a hydrogen bond with the ligand (Figure 2). In our simulations, the loop containing Tyr485 moved away from the ligand. In unconstrained simulations of WT GLB1, the most significant motions were coordinated interdomain motions. In unconstrained simulations of p.Asn490del, we observed the same type of interdomain motions, but their magnitude was diminished and less coordinated. Additionally, the motion of the catalytic loop was significantly greater than was observed in WT. Collective protein motions are typically related to function, which have been validated through functional‐structural studies in the literature (Caulfield & Medina‐Franco, 2011; Caulfield et al., 2012; Fiesel et al., 2014; Kayode et al., 2010; Puschmann et al., 1998; Zhang et al., 2011). The loss of collective motion, as we have observed for p.Asn490del, is likely to have a substantial impact on protein function. Thus, our simulations indicate a potential mechanism for the observed poor enzymatic activity—altered ligand interactions and loss of coordinated motions.

## 5 DISCUSSION

GM1 gangliosidosis is a rare disorder with an incidence of approximately 1:100,000–1:200,000 live births. Because of its rarity and the overlapping symptoms with the other neurodegenerative disorders, GM1 gangliosidosis is likely underdiagnosed (Brunetti‐Pierri & Scaglia, 2014). While it is known that accumulation of GM1 ganglioside and other glycoconjugates as a result of β‐galactosidase deficiency is responsible for the disease phenotype, the exact pathomechanism is still not completely understood and is an active area of research (Brunetti‐Pierri & Scaglia, 2014). Substrate reduction treatment has been proposed to halt the progression of the disease for individuals who have residual levels of β‐galactosidase activity. Iminosugar drugs have shown potential in reducing the storage of glycosphingolipids in Glb1‐deficient mice, resulting in improved survival (Elliot‐Smith et al., 1993; Kasperzyk et al., 2017). The iminosugar Miglustat, which has been approved for the treatment of Gaucher disease type I, was found to reverse disease progression in juvenile/adult GM1 gangliosidosis (Deodato et al., 2012). More recently, injections of adeno‐associated viral vectors (AAV9, AAVrh8) encoding GLB1 have shown success in increasing GLB1 activity, reducing symptoms, and extending lifespan in mice with GM1 gangliosidosis (Deodato et al., 1991; Weismann et al., 2007). This form of gene therapy has been extended to larger animal models with promising results paving the way for future clinical trials in humans (Gray‐Edwards et al., 2017).

We determined that the proband's unusual clinical presentation was due in part to her premature birth. The retinopathy of prematurity and bronchopulmonary dysplasia seen in the proband are obvious consequences of incomplete development (Crump, Winkleby, Sundquist, & Sundquist, 2014). Oral aversion requiring gastrostomy has been reported in infants having gastroschisis (Overcash et al., 2015). Use of gastrostomy to combat dysphagia and prevent aspiration resulting from GM1/GM2 gangliosidosis is also a common practice (Jarnes Utz et al., 2010).

The proband's coarse facial features, dysostosis multiplex, spinal deformities, psychomotor regression, hepatomegaly, dysphagia, apnea, cardiac defects, and generalized hypotonia are consistent with a diagnosis of GM1 gangliosidosis (Caciotti et al., 2017). While it would appear that her tonic–clonic seizures were precipitated by intraventricular hemorrhage secondary to preterm birth, seizures are also a common complication of GM1 gangliosidosis. Her joint stiffness and short limbs likely stemmed from her skeletal abnormalities, another complication of GM1 gangliosidosis.

Based on the family history, it is likely that the proband's parents are both carriers of the in‐frame deletion. Uniparental disomy (UPD) and consanguinity were ruled out by microarray. The proband has no other full siblings and has five maternal and four paternal half siblings. Carrier status of the parents and children could not be determined as they were lost to follow‐up. The variant has been reported in dbSNP (rs780232995) and gnomAD and has a minor allele frequency (MAF) of 0.00005811% in the Latino population (Lek et al., 2004). It is possible that this variant is more common in Latino populations given the ethnicity of both parents.

We used molecular modeling and dynamics simulations to generate an atomic mechanism for how the p.Asn490del might alter β‐galactosidase enzymatic activity. Predictions of this model could be confirmed using spectroscopic techniques. Also, there is the potential for molecular modeling to inform the underlying mechanisms of other metabolic conditions caused by pathogenic mutations in *GLB1*. Looking ahead, studies to examine flexible modes with advance modeling techniques coupled with experimental work to tease out putative binding sites, mutagenic regions of interest, countermutations, and protein stabilities would benefit the basic science and help accelerate drug discovery for this target, which has been successful previously (Li, Caulfield, Qiu, Copland, & Tun, 2013; López‐Vallejo et al., 1991; Puschmann et al., 1998; Zhang et al., 2011, 2014). These additional variants could be simulated to predict altered conformations and computational docking of different ligands could be used to determine which ligands are most strongly affected by each variant, thus aiding drug design. We believe that molecular modeling fills an important role in generating additional data and hypotheses for functional studies—critical steps in the evaluation of genetic variants identified by molecular testing.

## AUTHOR CONTRIBUTION

All authors made substantial contributions to the work including design, acquisition, analysis, or interpretation of data and in drafting of the work or revising it critically for important intellectual content. All authors gave final approval of the version to be published and agreed to be accountable for all aspects of the work in ensuring that questions related to the accuracy or integrity of any part of the work are appropriately investigated and resolved.

## DECLARATIONS

Compliance with Ethics Guidelines: The authors have no conflicts of interest to disclose.

Consent for Publication & Informed Consent: All procedures followed were in accordance with the ethical standards of the responsible committee on human experimentation (institutional and national) and with the Helsinki Declaration of 1975, as revised in 2000 (5). Informed consent was obtained from all patients included in the study.

Availability of Data & Materials: Datasets and materials are detailed in manuscript.
